# A Lamb Wave Wavenumber-Searching Method for a Linear PZT Sensor Array

**DOI:** 10.3390/s19194166

**Published:** 2019-09-25

**Authors:** Bin Liu, Tingzhang Liu, Jianfei Zhao

**Affiliations:** 1 School of Mechatronic Engineering and Automation, Shanghai University, Shanghai 200444, China; khqlb@shu.edu.cn (B.L.); jfzhao@shu.edu.cn (J.Z.); 2 Department of Quartermaster and Aviation POL, Air Force Logistics College, Xuzhou 221000, China

**Keywords:** Structural health monitoring, Lamb wave, wavenumber, linear PZT sensor array

## Abstract

In this paper, a wavenumber–searching method based on time-domain compensation is proposed to obtain the wavenumber of the Lamb wave array received signal. In the proposed method, the time-domain sampling signal of the linear piezoelectric transducer (PZT) sensor array is converted into a spatial sampling signal using the searching wavenumber. The two–dimensional time-spatial-domain Lamb wave received signal of the linear PZT sensor array is then converted into a one-dimensional synthesized spatial sampling signal. Further, the sum of squared errors between the synthesized spatial sampling signal and its Morlet wavelet fitting signal is calculated at each searching wavenumber. Finally, the wavenumber of the Lamb wave array received signal is obtained as the searching wavenumber corresponding to the minimum error. This method was validated on a 2024-T3 aluminum alloy. The validation results showed that the proposed method can successfully obtain the wavenumber of the Lamb wave array received signal, whose spatial sampling rate does not satisfy the Nyquist sampling theorem; the wavenumber error does not exceed 2.2 rad/m. Damage localization based on the proposed method was also validated on a carbon fiber composite laminate plate, and the maximum damage localization error was no more than 2.11 cm.

## 1. Introduction

Structural health monitoring (SHM) techniques use sensors integrated in a structure to monitor its overall real–time health status. This can enhance structure functionality, improve safety and reliability, reduce maintenance costs, and lengthen the service life; therefore, SHM has been studied for several industrial applications [1,2]. Among the various SHM techniques, guided Lamb wave-based SHM technology has attracted interest because it is sensitive to small-scale damage with a long detection range; it can also be applied in both active and passive monitoring [3,4,5,6,7,8]. In most applications, guided Lamb wave is generated and received by piezoelectric transducer (PZT) glued to the test specimen [6,7,8]. Many PZT sensor and Lamb wave-based damage monitoring methods have been studied, including the delay-and-sum [9,10,11], time reversal focusing–based [12,13,14], probability-based diagnostic [15,16,17], ultrasonic phased array [18,19,20], and multiple signal classification [21,22,23,24] imaging methods. In these methods, damage is identified and characterized by analyzing the characteristics of the damage monitoring signal in the time and/or frequency domain after the damage affects the Lamb wave scattering, such as time–of–flight, amplitude and energy. However, the Lamb wave has multimodal and non-linearly dispersed characteristics, which cause difficulties in extracting the damage scattering signal [25,26,27]. Because the different modes of the Lamb wave signal have different propagation characteristics, the damage location error becomes larger and even more difficult to locate.

Therefore, several authors have attempted to analyze and process the damage monitoring signal in the spatial–wavenumber domain. Distinction of the Lamb wave modes in the spatial-wavenumber domain is more efficient than in the time-frequency domain. Kannajosyula et al. [28] presented expressions representing the wavenumber spectra of annular phased array transducers for elastic guided wave mode selection. McKeon et al. [29] suggested a frequency–wavenumber–domain baseline subtraction technique to improve the lower limit of notch depth detection in plates. Cai et al. [30] improved the spatial resolution of the delay-and-sum damage imaging method by compensating for the dispersion of the Lamb wave signal using linearly dispersive signal construction with measured relative wavenumber curves. Zhu et al. [13] proposed a time-reversal technique in the frequency-wavenumber-domain using scattered flexural plate waves for fast identification of multiple damage sites. Rogge and Leckey [31] presented a local Fourier-domain analysis method for processing guided wave field data to estimate spatially dependent wavenumber values, which are related to thickness through dispersion relationships that are dependent on the depth of void-like defects. Tian and Yu [32] studied Lamb wave mode decomposition using decomposition filters designed by the dispersion curves in frequency-wavenumber coordinates. Purekar et al. [33] adopted a spatial-wavenumber filter to determine the damage direction based on the wavenumber curve. Qiu et al. [34,35,36,37] studied a spatial-wavenumber filter damage imaging method based on the wavenumber of the damage scattering signal projected at the linear PZT sensor array. At present, the wavenumber has importance in spatial-wavenumber-domain signal processing, similar to the importance of signal frequency in time-frequency-domain signal processing.

Lamb wave propagation in a plate involves oscillations in time as well as in space. The signal wavenumber represents its spatial vibration frequency. Accurate determination of the signal wavenumber is the basis of signal processing in the spatial-wavenumber domain. At present, four main methods for calculating the wavenumber of Lamb wave signal are used:

(1) Theoretical modeling method [38,39,40,41]: According to the Rayleigh-Lamb equation, the wavenumber of the Lamb wave signal can be calculated using the material parameters of the structure. However, for a complex structure or unknown material parameters, the wavenumber cannot be calculated.
(2) Phase-unwrapping method [42,43]: The excitation signal and the received signal of Lamb wave are separately phase–unwrapped to obtain the phase at the center frequency of the signal. Then, according to the wavenumber definition formula, the wavenumber of the Lamb wave can be calculated using the propagation distance. However, for unknown excitation signal or propagation distance of the Lamb wave, or for complex signal components, this method is difficult to use.
(3) Fourier-transform method [44,45,46]: According to the definition of the wavenumber, the wavenumber of Lamb wave can be obtained by the Fourier transform of the Lamb wave spatial sampling signal. This method generally requires a higher spatial sampling rate and longer spatial sampling length according to the properties of the Fourier transform. Therefore, this method is generally used to process Lamb wave spatial signals collected by scanning laser Doppler vibrometer (SLDV). However, it is inappropriate for use in online damage monitoring.
(4) Spatial-wavenumber filter method [34,35,36,37,47]: A series of spatial-wavenumber filters with different central wavenumbers are used to filter the spatial sampling signal of the Lamb wave. When the scanning wavenumber is equal to the wavenumber of the Lamb wave spatial sampling signal, the Lamb wave spatial sampling signal is able to pass the spatial–wavenumber filter. The wavenumber of the Lamb wave spatial sampling signal is then obtained. The spatial sampling rate of the linear PZT sensor array should satisfy the Nyquist sampling theorem in this method.

**Figure 1:**
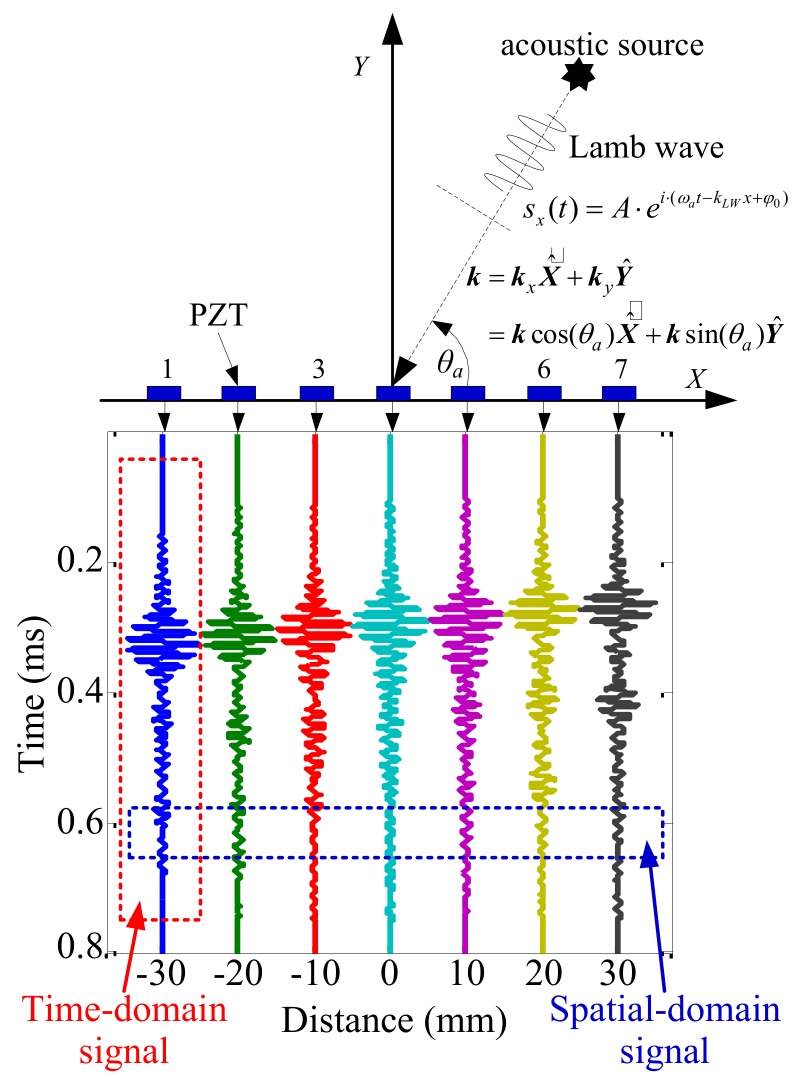
Lamb wave received signal of the linear PZT sensor array.

In previous spatial-wavenumber filter studies [34,35,36,37], the Lamb wave array received signal contains two parts—the time-domain signal and spatial-domain signal—as shown in Figure 1. It was easy to improve the temporal sampling frequency of the linear PZT sensor array. However, the spatial sampling rate of the linear PZT sensor array was limited by the PZT sensor size, so it was difficult to improve the spatial precision. In this study, a wavenumber searching method based on time–domain compensation is proposed to obtain the wavenumber of the Lamb wave array received signal. This method only requires that the temporal sampling frequency satisfies the Nyquist sampling theorem; the spatial sampling rate does not need to satisfy the Nyquist sampling theorem. According to the propagation formula of the Lamb wave and the searching wavenumber, the time-domain sampling signal of the linear PZT sensor array is converted into spatial sampling signal. The sum of squared errors between the spatial sampling signal and its Morlet wavelet fitting signal is then calculated. Finally, the wavenumber of the Lamb wave array received signal is obtained as the searching wavenumber corresponding to the minimum error. According to the authors’ previous studies [34,35], damage can be localized using the Lamb wave wavenumber-searching method and cruciform PZT sensor array.

The rest of the paper is organized as follows: The basic principle of the Lamb wave wavenumber-searching method is introduced in Section 2. In Section 3, the method of damage localization based on the Lamb wave wavenumber-searching method and the cruciform PZT sensor array is described. In Section 4, the Lamb wave wavenumber-searching method is validated on an aluminum plate. In Section 5, the damage localization method based on the Lamb wave wavenumber-searching method and cruciform PZT sensor array is validated on a carbon fiber composite laminate plate. Finally, conclusions are stated in Section 6.

## 2. The Lamb Wave Wavenumber-Searching Method

### 2.1. Theoretic Foundations of the Method

In a thin, isotropic, and homogeneous plate, the Lamb wave, regardless of mode, can generally be described by the following expression:

(1) $$s_{x}(t)=A\cdot e^{i\cdot (\omega_{a}t-k_{LW}x+\varphi_{0})}$$

where *x* is the propagation distance of the Lamb wave, *t* is the propagation time, *A* is the vibration amplitude, *e* is the Euler’s number, *i* is the imaginary unit, *ω_a_* is the angular frequency of the Lamb wave, *k_LW_* is the wavenumber of the Lamb wave, and *φ*_0_ is the initial phase of the Lamb wave.

It can be seen from Equation (1) and Figure 1 that the Lamb wave has components that vary harmonically in both time and space. According to the description of Lamb wave propagation, the propagation distance of a specific phase in time *t* is:

(2) $$x=t\cdot c_{p}=\frac{t\cdot \omega_{a}}{k_{LW}}$$

where *c_p_* is the phase velocity of the Lamb wave. 

Equation (2) indicates that the time-domain sampling can be converted into spatial-domain sampling using the central frequency and wavenumber of the Lamb wave.

When the temporal sampling frequency satisfies the Nyquist sampling theorem:

(3) $$\omega_{s}=\frac{2\pi }{\Delta t}>2\omega_{a}$$

where *ω_s_* is the temporal sampling frequency of the sensor and △*t* is the time-domain sampling interval of the sensor; then, the spatial sampling interval Δ*x* of the sensor is:

(4) $$\Delta x=\Delta t\cdot c_{p}=\frac{\Delta t\cdot \omega_{a}}{k_{LW}}$$

and the spatial sampling rate *k_s_* of the sensor is:

(5) $$\left(k_{s}=\frac{2\pi }{\Delta x}=\frac{2\pi }{\Delta t}\cdot \frac{k_{LW}}{\omega_{a}}\right)>\left(2\omega_{a}\cdot \frac{k_{LW}}{\omega_{a}}=2k_{LW}\right)$$

Equation (5) indicates that if the sampling signal satisfies the Nyquist sampling theorem in the time-domain, its conversion into the spatial-domain using the wavenumber and central frequency will also satisfy the Nyquist sampling theorem. Therefore, we can use high temporal sampling rate signals to compensate for low spatial sampling rate signals.

### 2.2. The Principle of the Method

**Figure 2:**
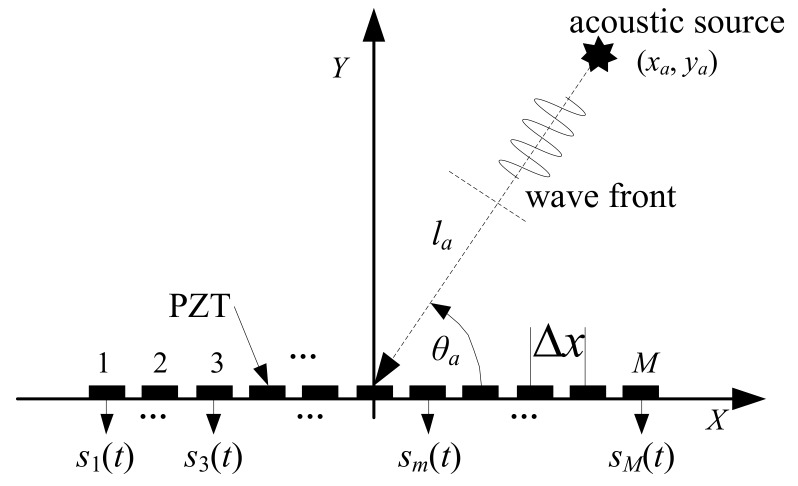
Spatial sampling of the Lamb wave by the linear PZT sensor array.

A linear PZT sensor array comprising *M* PZT sensors is placed on the surface of the monitored structure, as shown in Figure 2. The PZT sensors are indexed as *m* = 1, 2,..., *M*, and *M* is assumed to be an odd number. A Cartesian coordinate system is constructed on the monitoring structure with the center point of the linear PZT sensor array set as the origin, and the linear PZT sensor array itself set as the *X*-axis. 

The distance between the centers of each pair of adjacent PZT sensors, or the spatial sampling interval of the linear PZT sensor array, is denoted Δ*x*. The spatial sampling rate of the linear PZT sensor array is 2*π*/Δ*x*. As shown in Figure 2, an acoustic source is located at (*x_a_*, *y_a_*). The direction (angle) and distance of the acoustic source relative to the linear PZT sensor array are *θ_a_* and *l_a_*, respectively. A narrowband-frequency Lamb wave with the central frequency *f_a_* and wavenumber *k_LW_*, respectively, is excited by the acoustic source. The angular frequency of the narrowband–frequency Lamb wave is *ω_a_* = 2*πf_a_*. All PZT sensors in the linear PZT sensor array are used to collect the Lamb wave simultaneously with the temporal sampling frequency *f_s_*, which satisfies the Nyquist sampling theorem *f_s_* > 2*f_a_*. Then, the Lamb wave received signals collected by the linear PZT sensor array in the time-spatial domain can be expressed as Equation (6). The central frequency of the Lamb wave received signal is also *f_a_*, and its wavenumber is *k_a_* = *k_LW_*·cos(*θ_a_*) [34,35,36,37]:

(6) $$S=\left[\begin{array}{ccccc} s_{1}(0) & \ldots & s_{m}(0) & \ldots & s_{M}(0)\\ \ldots & & \ldots & & \ldots \\ s_{1}(t) & \ldots & s_{m}(t) & \ldots & s_{M}(t)\\ \ldots & & \ldots & & \ldots \\ s_{1}(T) & \ldots & s_{m}(T) & \ldots & s_{M}(T) \end{array}\right]$$

where *s*_1_(0) is the sampling value of PZT 1 at *t* = 0 (starting time), *s*_1_(*t*) is the sampling value of PZT 1 at time *t*, *s*_1_(*T*) is the sampling value of PZT 1 at time *T*, *s_m_*(0) is the sampling value of PZT *m* at *t* = 0 time, *s_m_*(*t*) is the sampling value of PZT *m* at time *t*, *s_m_*(*T*) is the sampling value of PZT *m* at time *T*, *s_M_*(0) is the sampling value of PZT *M* at *t* = 0, *s_M_*(*t*) is the sampling value of PZT *M* at time *t*, and *s_M_*(*T*) is the sampling value of PZT *M* at time *T*.

According to Equation (2), the time-domain sampling signal of each PZT sensor can be converted into spatial-domain sampling signal using the central frequency and wavenumber of the Lamb wave received signal and the position of the PZT sensor:

(7) $$S\prime =\left[\begin{array}{ccccc} s_{1}(x_{1-0}) & \ldots & s_{m}(x_{m-0}) & \ldots & s_{M}(x_{M-0})\\ \ldots & & \ldots & & \ldots \\ s_{1}(x_{1-t}) & \ldots & s_{m}(x_{m-t}) & \ldots & s_{M}(x_{M-t})\\ \ldots & & \ldots & & \ldots \\ s_{1}(x_{1-T}) & \ldots & s_{m}(x_{m-T}) & \ldots & s_{M}(x_{M-T}) \end{array}\right]$$

In Equation (7), *s_m_*(*x_m_*_–*t*_) is the spatial sample value of the spatial sampling position *x_m_*_–*t*_. Then, Equation (7) can be rewritten as:

(8) $$S\prime =[s_{1}(x_{1-0}),\ldots ,s_{1}(x_{1-T}),\ldots ,s_{m}(x_{m-0}),\ldots ,s_{m}(x_{m-T}),\ldots ,s_{M}(x_{M-0}),\ldots ,s_{M}(x_{M-T})]$$

The spatial samples *s_m_*(*x_m_*_–*t*_) are sorted according to the spatial sampling position *x_m_*_–*t*_ from small to large. If the spatial sampling positions are the same, their spatial sample values are averaged. Finally, the two–dimensional time-spatial-domain Lamb wave received signal *S* is converted into one-dimensional spatial-domain Lamb wave received signal *G*:

(9) $$G=[g(z_{1},\ldots ,g(z_{r},\ldots ,g(z_{R}]$$

where *z_r_* is the synthesized spatial sampling position, *g*(*z_r_*) is the spatial sampling value at the spatial position *z_r_*, and *R* is the length of the synthesized spatial sampling signal.

In order to facilitate the subsequent Morlet wavelet transform, the average spatial sampling interval Δ*z*′ is calculated first, because the synthesized spatial sampling positions are generally non-uniformly spaced:

(10) $$\Delta z^{\prime }=\frac{\sum_{r=2}^{R}\left[z_{r}-z_{\left(r-1\right)}\right]}{R-1}$$

The uniformly spaced synthetic spatial sampling position *z*′ is generated according to the average spatial sampling interval Δ*z*′ and the length *R* of the synthesized spatial sampling signal. The corresponding synthetic spatial sampling signal *G*′ is linearly interpolated from the original synthesized spatial sampling signal *G*. The Morlet wavelet can be used to remove signal noise and extract specific narrowband–frequency signals from a wideband–frequency signal [34,37,48]. Using the uniformly spaced synthetic spatial sampling position *z*′ and the wavenumber *k*, the Morlet wavelet fitting waveform *G*″ can be obtained by Morlet wavelet transform of the synthetic spatial sampling signal *G*′.

**Figure 3:**
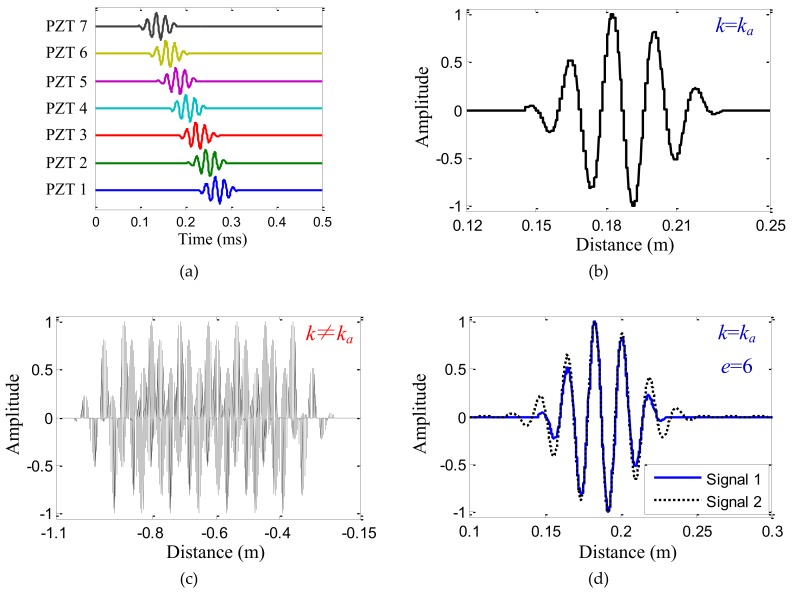

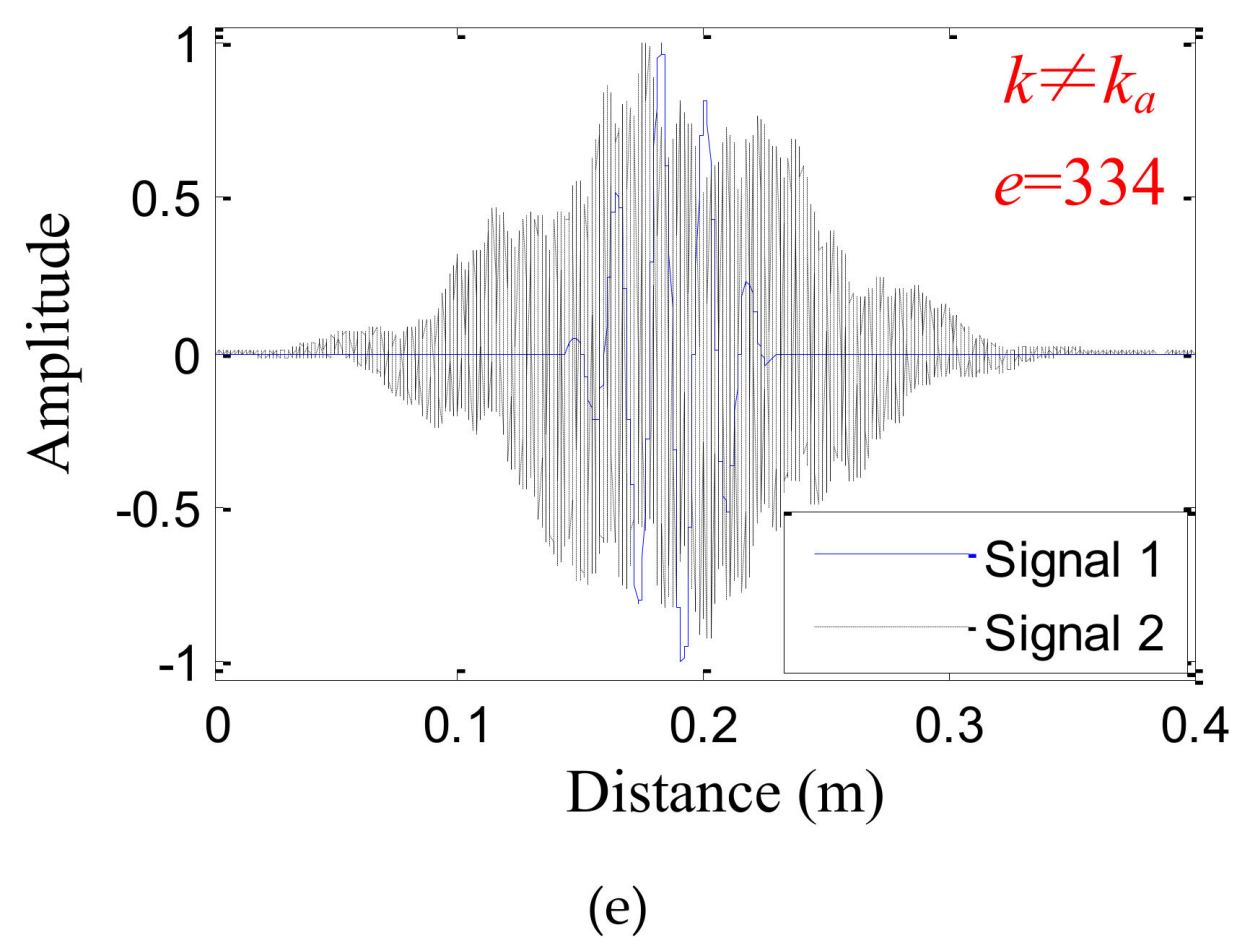
Schematics of the Lamb wave wavenumber–searching method. (**a**) Lamb wave received signals collected by the linear PZT sensor array; (**b**) The synthetic spatial sampling signal *G*′ with the wavenumber *k* = 342.5 rad/m = *k_a_*; (**c**) The synthetic spatial sampling signal *G*′ with the wavenumber *k* = −100 rad/m ≠ *k_a_*; (**d**) The Morlet wavelet fitting waveform *G*″ with the wavenumber *k* = 342.5 rad/m = *k_a_*; (**e**) The Morlet wavelet fitting waveform *G*″ with the wavenumber *k* = −100 rad/m ≠ *k_a_*.

Based on the analysis in Section 2.1, the synthetic spatial sampling signal *G*′ is the spatial sampling signal of the Lamb wave for the wavenumber *k* = *k_a_*, as shown in Figure 3b. Otherwise, the synthetic spatial sampling signal *G*′ is messy, as shown in Figure 3c. In Figure 3, the central frequency and wavenumber of the Lamb wave received signal are 50 kHz and 342.5 rad/m. “Signal 1” is the spatial sampling signal of the Lamb wave. “Signal 2” is the Morlet wavelet fitting waveform *G*″. According to the properties of the Morlet wavelet transform, when the wavenumber of the Morlet wavelet fitting waveform *G*″ is equal to that of the synthetic spatial sampling signal, the sum of the squared error, *e*, calculated by Equation (11), between the Morlet wavelet fitting waveform *G*″ and the synthetic spatial sampling signal *G*′ is small, as shown in Figure 3d. Otherwise, the sum of the squared error *e* is large, as shown in Figure 3e.

(11) $$e=\sum {\left(G^{\prime \prime }-G^{\prime }\right)}^{2}$$

In summary, the wavenumber searching range is set to [*k*_1_, *k_N_*] and the resolution is set to Δ*k*. Then, the sum of squared error *e* at each searching wavenumber is obtained. Finally, the wavenumber at the minimum value of *e* is considered the best match to the wavenumber *k_b_* of the Lamb wave received signal.

In order to ensure the accuracy of calculation and the validity of compensation, the length of the spatial-domain sampling signal converted from the time-domain sampling signal must be longer than that of the linear PZT sensor array:

(12) $$\frac{T\cdot \omega_{a}}{k}>(M-1)\Delta x,$$

Equation (12) indicates that the maximum searching wavenumber should be:

(13) $$k_{N}<\frac{T\cdot \omega_{a}}{(M-1)\Delta x},$$

In practical application, the sum of squared error *e* fluctuates greatly caused by various factors, which easily causes misjudgment. Therefore, the moving average method is used in this paper to smooth *e*.

**Figure 4:**
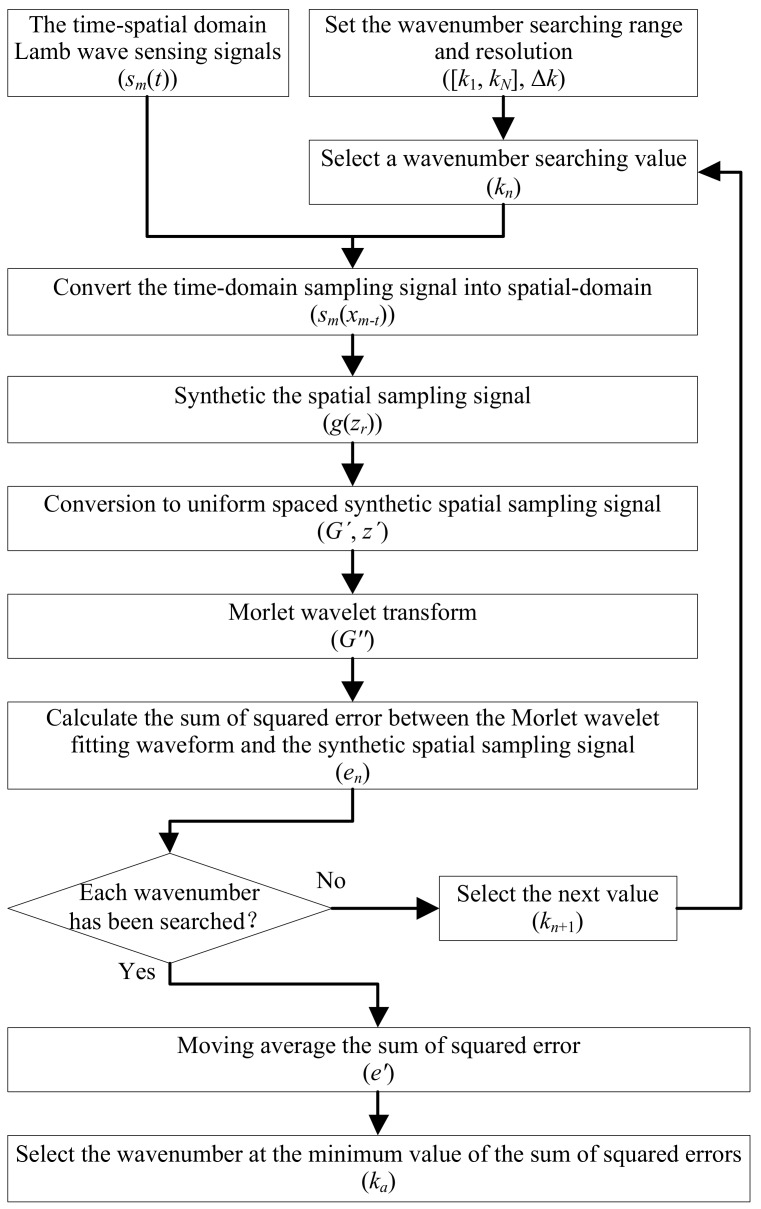
The implementation process of the Lamb wave wavenumber-searching method.

Based on the above analysis, the implementation process of the Lamb wave wavenumber–searching method based on time-domain compensation is shown in Figure 4.

## 3. Damage Localization Based on the Lamb Wave Wavenumber–Searching Method and Cruciform PZT Sensor Array

Based on the authors’ previous studies [34,35], structural damage can be localized using the Lamb wave wavenumber-searching method and the cruciform PZT sensor array. The cruciform PZT sensor array comprises two linear PZT sensor arrays numbered No. 1 and No. 2. The center point of the cruciform PZT sensor array is set as the origin point. The *X*-axis and *Y*-axis are set along the directions of the No. 1 and No. 2 linear PZT sensor arrays, respectively. Direction *θ_a_* of the damage can then be obtained by the wavenumber of the damage scattering signal projected at the *X*-axis and *Y*-axis (No. 1 and No. 2 linear PZT sensor arrays), as studied in detail in the previous references [34,35].

According to the Lamb wave propagation description (Equation (1)) and the principle of phased array, the envelope of the synthesized signal of the cruciform PZT sensor array is expressed as Equation (14) using the received signal wavenumbers of No. 1 and No. 2 linear PZT sensor arrays. The time corresponding to the maximum value of the envelope is the arrival time *t_a_* of the damage scattering signal. The start time *t_e_* of the excitation signal is obtained by the Shannon wavelet transformation of the excitation signal [35,36]. Then, the damage distance *l_a_* can be calculated as Equation (15) using the Lamb wave velocity.

(14) $$H\left(t\right)=\left|\frac{1}{M}\sum_{m=1}^{M}\left(e^{ik_{1}x_{m}}S_{1-m}+e^{ik_{2}y_{m}}S_{2-m}\right)\right|$$

where *k*_1_ is the wavenumber of the Lamb wave received signal of the No. 1 PZT sensor array, *k*_2_ is that of the No. 2 array.

(15) $$l_{a}=\frac{c_{g}(t_{a}-t_{e})}{2}$$

where *c_g_* is the Lamb wave velocity. This yields the localized damage position (*θ_a_*, *l_a_*).

## 4. Validation Experiment of the Lamb Wave Wavenumber-Searching Method

### 4.1. Experimental Setup

**Figure 5:**
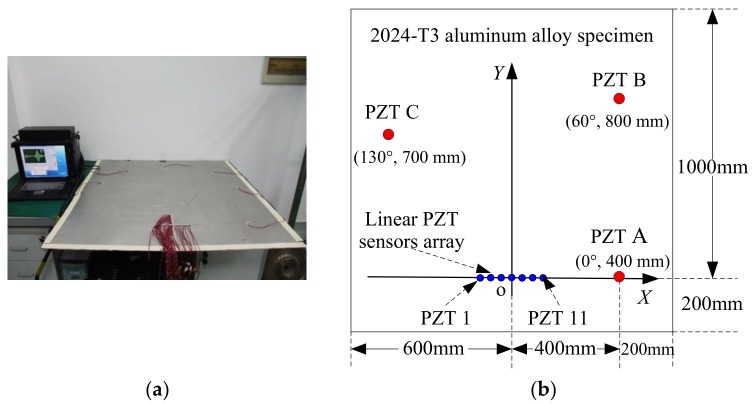
Experimental setup for the Lamb wave wavenumber-searching method. (**a**) Experiment setup; (**b**) Illustration of the linear PZT sensor array, acoustic sources, and coordinate system.

Because the wavenumber calculation methods for Lamb wave propagating in aluminum plate are relatively mature, the Lamb wave wavenumber-searching method is validated on an aluminum plate, and the results are compared with those from an existing method. The Lamb wave wavenumber-searching method demonstration system is shown in Figure 5, comprising the 2024-T3 aluminum alloy specimen, a linear PZT sensor array bonded to the plate surface, and an integrated SHM system.

The dimensions of the 2024-T3 aluminum alloy specimen are 1200 mm × 1200 mm × 2 mm (length × width × thickness). The mechanical properties of the 2024-T3 aluminum alloy are shown in Table 1.

The linear PZT sensor array comprises 11 PZT sensors with a spacing of 18 mm. The type, diameter, and thickness of the PZT sensor are PZT-5A, 8 mm, and 0.48 mm, respectively. The PZT sensors in the linear PZT sensor array are labeled as PZT 1, PZT 2, …, PZT 11, respectively, from left to right. The central point and axis of the linear PZT sensor array are taken as the coordinate origin and the *X*-axis, respectively. This constructs a two-dimensional rectangular coordinate system in the specimen, as shown in Figure 5. In addition, another three PZT sensors used as the actuator for Lamb wave excitation are bonded at (0°, 400 mm), (60°, 800 mm), and (130°, 700 mm), and labeled as PZT A, PZT B, and PZT C, respectively. The spatial sampling rate *k_s_* of the linear PZT sensor array is:

(16) $$k_{s}=\frac{2\pi }{\Delta x}=\frac{2\pi }{0.018}\approx 349(\mathrm{rad}/m)$$

According to the Nyquist sampling theorem, the corresponding maximum wavenumber processing capability of the linear PZT sensor array is 174 rad/m.

The integrated SHM system developed by Yuan of the Nanjing University of Aeronautics and Astronautics is adopted as the Lamb wave excitation and acquisition equipment [49]. In this experimental verification, the excitation signal is a modulated five-cycle sine burst, as shown in Equation (17) [50]. The center frequency of the excitation signal ranges from 30 kHz to 70 kHz with an interval of 5 kHz, and the amplitude is ± 70 V. The sampling rate is 10 MHz and the sampling length is 10000 samples, including 1000 pre-samples. The trigger voltage is 6 V. Lamb waves with different central frequencies are excited by the three actuators in turn. The Lamb wave propagating in the 2024-T3 aluminum alloy specimen is collected by the linear PZT sensor array:

(17) $$s\left(t\right)=0.5\sin\left(2\pi f_{a}t\right)\left(H\left(t\right)-H\left(t-\frac{J}{f_{a}}\right)\right)\left(1-\cos\left(\frac{2\pi f_{a}t}{J}\right)\right)$$

where *H*(*t*) denotes the Heaviside function, *J* is the number of modulated cycles of the sine burst.

### 4.2. Theoretical Wavenumber Calculation

According to the Rayleigh-Lamb equation, the wavenumber of the Lamb wave of each central frequency is calculated using the material properties of the 2024-T3 aluminum. Only *A*_0_ and *S*_0_ modes exist in the Lamb wave at low central frequency (30–70 kHz), and the amplitude of the *A*_0_ mode is much higher than that of the *S*_0_ mode. Thus, only the wavenumber of the *A*_0_ mode is calculated.

Anti-symmetric mode of the Rayleigh-Lamb equation:

(18) $$\frac{\tan k_{t}b}{\tan k_{l}b}=-\frac{{\left(k_{0}^{2}-k_{t}^{2}\right)}^{2}}{4k_{0}^{2}k_{l}k_{t}}$$

The longitudinal wavenumber *k_l_* and transverse wavenumber *k_t_* are calculated as:

(19) $$k_{l}^{2}={\left(\frac{\omega }{C_{l}}\right)}^{2}-k_{0}^{2},\;k_{t}^{2}={\left(\frac{\omega }{C_{s}}\right)}^{2}-k_{0}^{2}$$

The longitudinal wave velocity *C_l_* and transverse wave velocity *C_s_* are calculated as:

(20) $$C_{l}=\sqrt{\frac{\lambda +2G}{\rho }},\;C_{s}=\sqrt{\frac{G}{\rho }}$$

The Lamé first parameter *λ* and second parameter (shear modulus) *G* are calculated as:

(21) $$\lambda =\frac{E*\mu }{\left(1+\mu )(1-2*\mu \right)},\;G=\frac{E}{2(1+\mu )}$$

where *k*_0_ denotes the horizontal wavenumber, *b* is one-half the plate thickness, *ω* is the angular frequency of the Lamb wave, *E* is Young’s modulus, *μ* is Poisson’s ratio, and *ρ* denotes density.

Assumed:

(22) $$B=\frac{\omega^{2}}{C_{l}^{2}}-k^{2},\;D=\frac{\omega^{2}}{C_{s}^{2}}-k^{2}$$

Equation (18) can be rewritten as:

(23) $$4\times \sqrt{-B}\times \sqrt{-D}\times (-i)\times k^{2}\times \tan(h\times \sqrt{D})=(k^{2}-D{)}^{2}\times (-i)\times \tan(h\times \sqrt{B}),$$

The frequency calculating range of 1–100 kHz is set with an interval of 1 kHz; the wavenumber calculating range is 0–1000 rad/m with an interval of 0.01 rad/m, and the error between the left-hand side and right-hand side of Equation (23) is 0.00001. Equation (23) is calculated by iterative computation using the material properties of the 2024-T3 aluminum given in Table 1. The theoretical calculated wavenumbers are listed in Table 2.

### 4.3. Typical Signal Analysis

**Figure 6:**
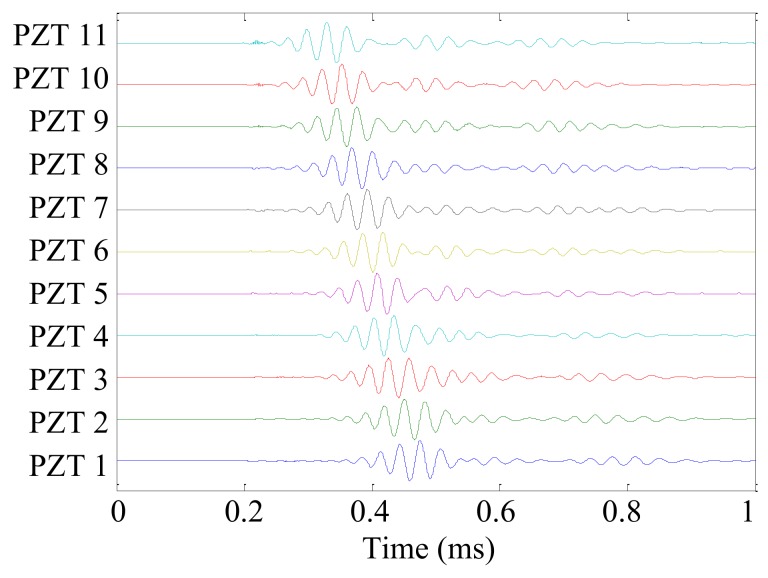
Lamb wave received signals *s_m_*(*t*) of PZT A with a center frequency of 30 kHz.

The Lamb wave received signal of PZT A with the center frequency of 30 kHz is used as an example to describe the process of signal analysis and processing in detail. The Lamb wave received signals *s_m_*(*t*) of PZT A are shown in Figure 6.

According to Equation (13) and the parameters of the experiment, the maximum searching wavenumber can be calculated.

(24) $$k_{N}<\frac{T\cdot \omega_{0}}{(M-1)\Delta x}=\frac{10000\times 10^{-7}\times 2\pi \times 30\times 10^{3}}{\left(11-1\right)\times 0.018}\approx 1047(\mathrm{rad}/m)$$

**Figure 7:**
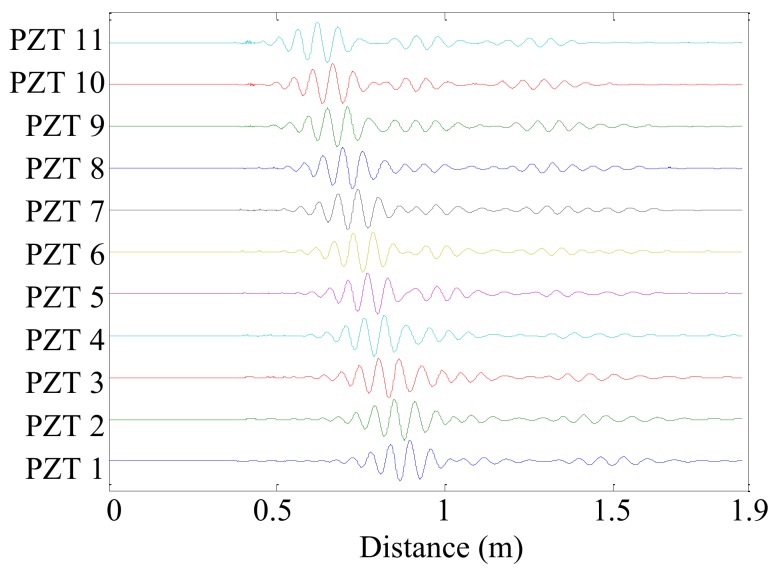
Converted spatial-domain sampling signal *s_m_*(*x_m_*_–*t*_) of each PZT sensor whose searching wavenumber is *k_n_* = 100 rad/m.

Then, the searching range of the wavenumber is set to [−600 rad/m, 600 rad/m] and the wavenumber resolution is set to Δ*k* = 0.1 rad/m, because the wavenumber of the Lamb wave with a center frequency of 70 kHz is below 500 rad/m. In each searching wavenumber, the time–domain sampling signal of each PZT sensor is converted into a spatial-domain sampling signal by Equation (2). Here, *k_n_* = 100 rad/m is selected as an example. The converted spatial-domain sampling signal *s_m_*(*x_m_*_–*t*_) of each PZT sensor is shown in Figure 7. In Figure 7, the spatial sampling point 0 m of each converted spatial-domain sampling signal refers to the position of the PZT sensor. In other words, the converted spatial-domain sampling signal of each PZT sensor begins at the sensor position.

**Figure 8:**
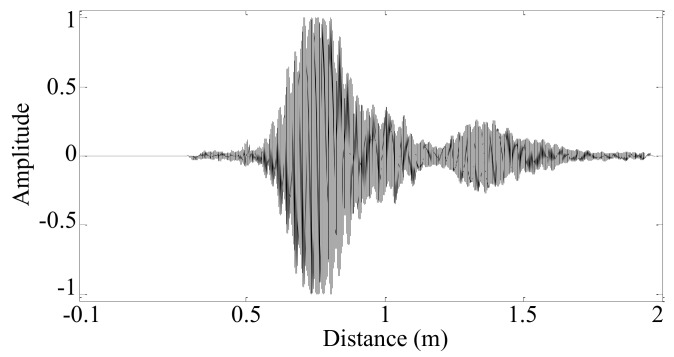
Synthesized spatial sampling signal *g*(*z_r_*).

The converted spatial-domain sampling signal *s_m_*(*x_m–t_*) of each PZT sensor is sorted according to the spatial sampling position *x_m_*_–*t*_ from small to large. If the spatial sampling positions are the same, their spatial sample values are averaged. Finally, the converted spatial-domain sampling signal of each PZT sensor is synthesized into a series of spatial sampling signals, as shown in Figure 8.

The average interval of spatial sampling position *z* is calculated as:

(25) $$\Delta z^{\prime }=\frac{\sum_{r=2}^{R}\left[z_{r}-z_{\left(r-1\right)}\right]}{R-1}=0.000037829m$$

**Figure 9:**
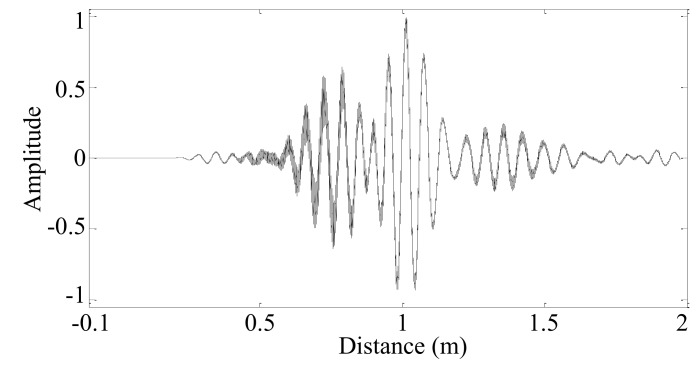
Morlet wavelet fitting waveform *G*″ whose wavenumber is *k_n_* = 100 rad/m.

Then, the uniformly spaced synthetic spatial sampling positions *z*′ can be obtained using the average spatial sampling interval Δ*z*′ and the length *R* of the synthesized spatial sampling signal. The corresponding synthetic spatial sampling signal *G*′ can be obtained by linear interpolation. Using the uniformly spaced synthetic spatial sampling position *z*′ and the wavenumber *k_n_* = 100 rad/m, the Morlet wavelet fitting waveform *G*″ can be obtained by the Morlet wavelet transformation of the synthetic spatial sampling signal *G*′, as shown in Figure 9. The sum of the squared error *e_n_* = 6839.9 between the Morlet wavelet fitting waveform and the synthetic spatial sampling signal can be calculated by Equation (11).

**Figure 10:**
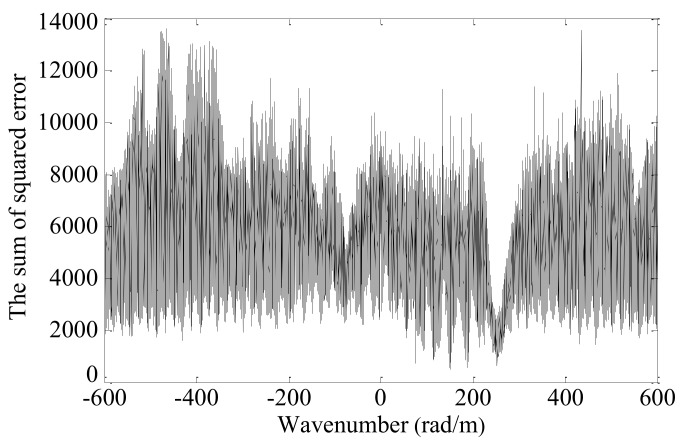
The sum of squared error *e_n_* at each searching wavenumber.

The sum of squared error *e_n_* between the Morlet wavelet fitting waveform and the synthetic spatial sampling signal are calculated in turn, as shown in Figure 10.

**Figure 11:**
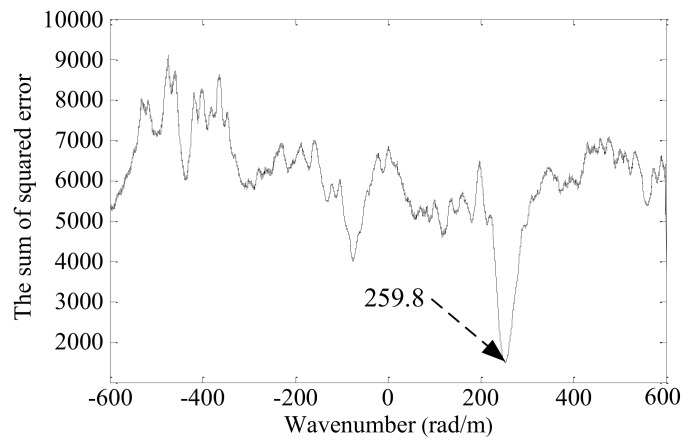
The smoothed sum of squared error.

The sum of squared error is smoothed by the moving-average method, as shown in Figure 11. Finally, the wavenumber of 259.8 rad/m at the minimum error value of 1483.5 is selected as the wavenumber of the Lamb wave received signal. The corresponding wavenumber searching error is −1.9 rad/m.

According to the Lamb wave received signal of PZT A with the center frequency of 30 kHz, the wavenumbers of the other Lamb wave actuator and central frequencies are compared with the theoretical wavenumbers, as shown in Table 3.

The experimental results show that the proposed Lamb wave wavenumber-searching method can be used to obtain the wavenumber of the Lamb wave received signals, whose spatial sampling rate does not satisfy the Nyquist sampling theorem. According to the Nyquist sampling theorem, the maximum wavenumber processing capability of the linear PZT sensor array is 174 rad/m, but the wavenumber of PZT A with the center frequency of 70 kHz is 410 rad/m in this experiment. The wavenumber error is no more than 2.2 rad/m. This is very useful for broadening the applicability of SHM based on linear PZT sensor array and spatial–wavenumber–domain signal processing.

## 5. Validation of the Damage Localization Method

### 5.1. Experimental Setup

**Figure 12:**
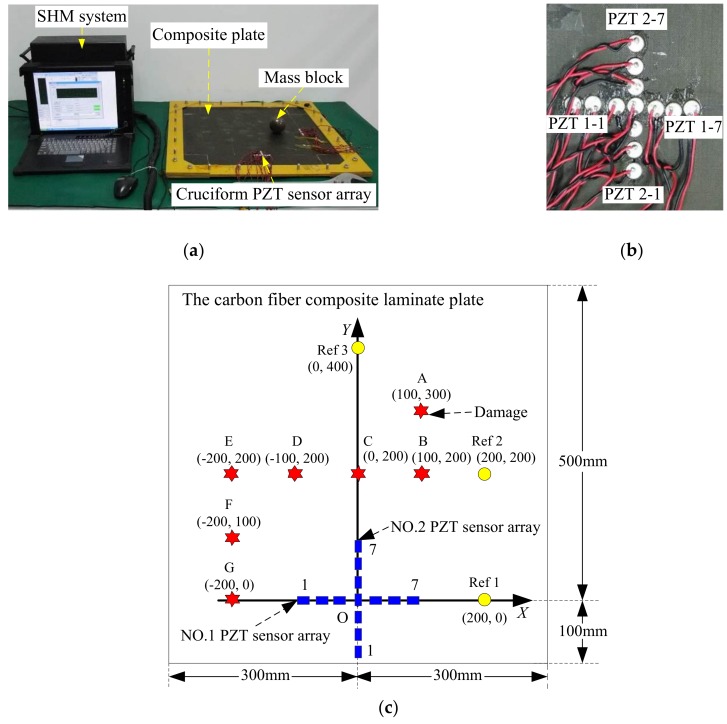
Experimental system for validating the damage localization method based on the Lamb wave wavenumber-searching method and cruciform PZT sensor array. (**a**) Experiment setup; (**b**) Cruciform PZT sensor array; (**c**) Illustration of the cruciform PZT sensor array placement and damage positions.

The validation experimental system shown in Figure 12 comprises a carbon fiber composite laminate plate, a cruciform PZT sensor array and an integrated SHM system.

The dimension of the carbon fiber composite laminate specimen is 600 mm × 600 mm × 2.25 mm (length × width × thickness). The composite plate comprises 18 single layers with the ply sequence of [45/0/−45/90/0/−45/0/−45/0/45/0/−45/90/0/−45/0/−45/0]. The material properties of each layer are shown in Table 4. 

Each linear PZT sensor array in the cruciform PZT sensor array comprises seven PZT sensors. The distance between the centers of each pair of adjacent PZT sensors is 10 mm. The PZT sensors in No. 1 array are labeled as PZT 1-1, PZT 1-2, …, PZT 1-7; those in No. 2 array are labeled as PZT 2-1, PZT 2-2, …, PZT 2-7. A PZT used as the actuator for Lamb wave excitation is placed at the center point of the cruciform PZT sensor array on the opposite side of the carbon fiber composite laminate plate. Another three reference PZT sensors labeled Ref 1, Ref 2, and Ref 3 are used to measure the Lamb wave group velocity. Their positions are shown in Figure 12c and Table 5.

The mass block shown in Figure 12a is used to change the local stiffness of the structure to simulate damage. In total, seven damage sites labeled A to G are simulated on the structure. The locations of these sites are shown in Table 5. The direction is defined according to the counterclockwise direction relative to the positive direction of the *X*-axis. The integrated SHM system is adopted to excite and acquire the Lamb wave signals. The excitation signal is the modulated five-cycle sine burst with the center frequency is 50 kHz and the amplitude is ± 70 V. The temporal sampling frequency is 10 MHz and the sampling length is 8000 samples, including 1000 pre-samples. The trigger voltage is 6 V. The experimental process is described as follows:

(1) The group velocity of Lamb wave was measured using the continuous complex Shannon wavelet transformation. The actuator was used to excite Lamb wave propagating on the composite plate, while the three reference PZT sensors were used to acquire the corresponding Lamb wave. For each reference PZT sensor, the group velocity was calculated as *c_g-_*_Ref 1_ = 1569.32 m/s, *c_g-_*_Ref 2_ = 1557.29 m/s, and *c_g-_*_Ref 3_ = 1485.65 m/s. Then, the average group velocity *c_g_* = 1537.42 m/s was obtained and used in damage localization.
(2) In the health state of the carbon fiber composite laminate plate, the Lamb wave signals of the cruciform PZT sensor array were acquired as health reference signals, *f*_HR_.
(3) Damage is created in each position and the corresponding Lamb wave signals of the cruciform PZT sensor array were acquired as the online monitoring signals, *f*_OM_.

### 5.2. Damage Localization Validation

**Figure 13:**
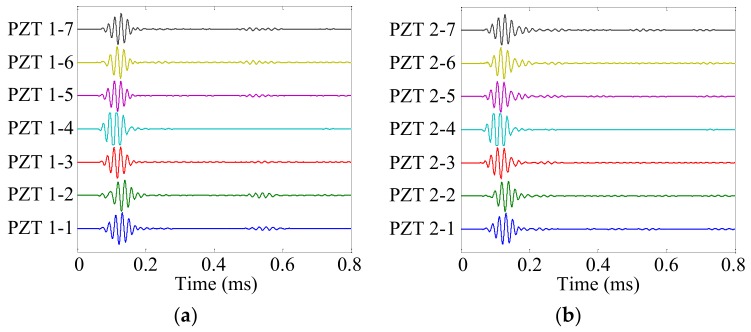
Health reference signals of the cruciform PZT sensor array. (**a**) No. 1 PZT sensor array; (**b**) No. 2 PZT sensor array.

**Figure 14:**
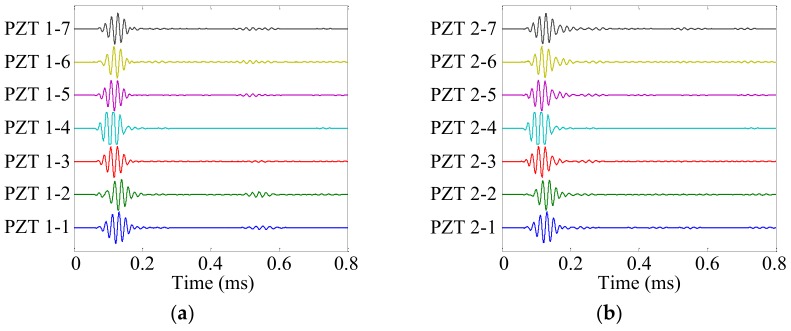
Online monitoring signals of the cruciform PZT sensor array. (**a**) No. 1 PZT sensor array; (**b**) No. 2 PZT sensor array.

**Figure 15:**
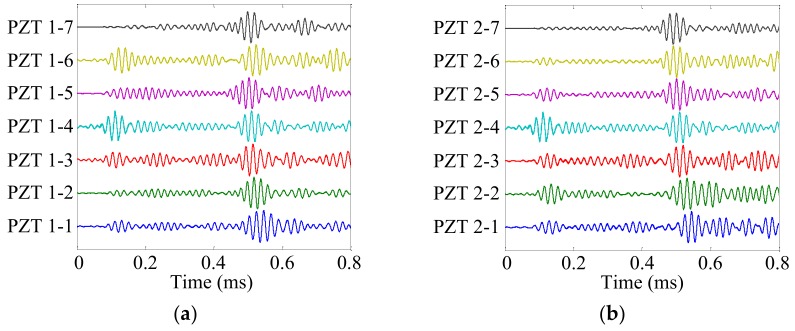
Damage scattering signals of the cruciform PZT sensor array. (**a**) No. 1 PZT sensor array; (**b**) No. 2 PZT sensor array.

Damage site A was selected to show the damage localization process. The health reference signals *f*_HR_ and online monitoring signals *f*_OM_ of the cruciform PZT sensor array are shown in Figure 13 and Figure 14, respectively. The damage scattering signals extracted by subtracting *f*_OM_ from *f*_HR_ are shown in Figure 15.

**Figure 16:**
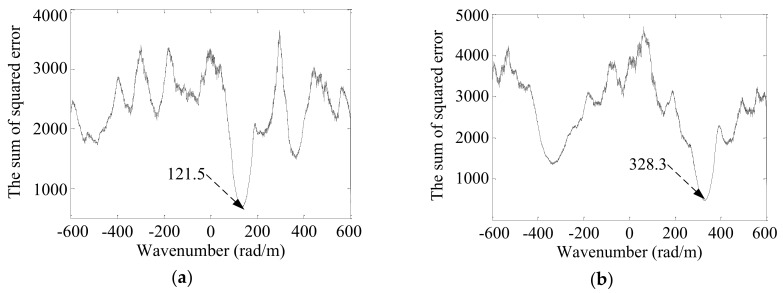
The sum of squared error of the cruciform PZT sensor array. (**a**) No. 1 PZT sensor array; (**b**) No. 2 PZT sensor array.

The Lamb wave wavenumber-searching method was applied to the damage scattering signals. The wavenumber searching range was set as [−600 rad/m, 600 rad/m] and the wavenumber searching interval was Δ*k* = 0.1 rad/m. Figure 16 shows the sum of squared error of the two linear PZT sensor arrays. The best-matching wavenumbers *k*_1_ = 121.5 rad/m and *k*_2_ = 328.3 rad/m are obtained from the two plots of *e*.

**Figure 17:**
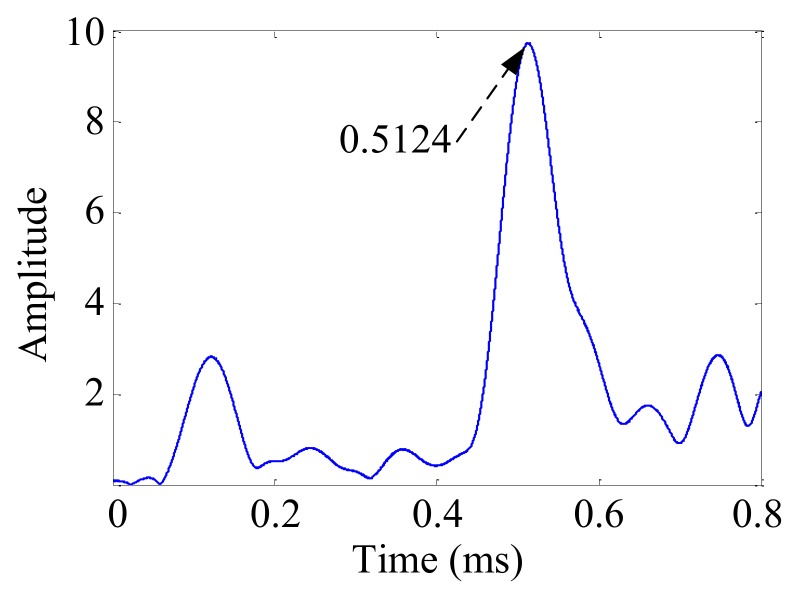
Envelope of the synthesized signal of the cruciform PZT sensor array.

**Figure 18:**
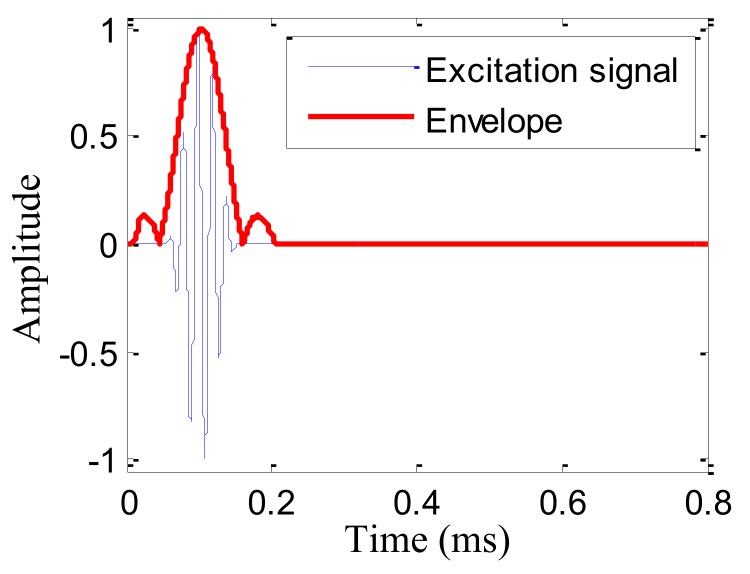
Excitation signal and start time.

According to our previous study [34,35], the damage direction *θ_a_* = 69.7° relative to the center point of the cruciform PZT sensor array can be obtained using the wavenumbers *k*_1_ and *k*_2_. According to Equation (14) and the wavenumbers *k*_1_ and *k*_2_, the arrival time *t_a_* = 0.5124 ms of the damage scattering signal could be obtained, as shown in Figure 17. The start time *t_e_* = 0.1032 ms of the excitation signal was obtained by the Shannon wavelet transformation of the excitation signal, as shown in Figure 18.

Then, the damage distance relative to the center point of the cruciform PZT sensor array *l_a_* = 314.6 mm was calculated according to Equation (15) and the Lamb wave velocity *c_g_* = 1537.42 m/s.

Finally, the damage position was obtained as (109.2 mm, 295.0 mm). The damage localization error was Δ*l* = 10.4 mm, as calculated by Equation (26):

(26) $$\Delta l=\sqrt{{\left(x_{a}-x_{D}\right)}^{2}+{\left(y_{a}-y_{D}\right)}^{2}},$$

where (*x_D_*, *y_D_*) is the actual position of the damage.

According to the damage localization process of damage site A, the damage localization results and errors of the seven damages are listed in Table 6. The table indicates that the damage localization results were in good agreement with the actual damage positions; the maximum damage localization error was no more than 2.11 cm.

## 6. Conclusions

This paper proposes a time-domain compensation-based Lamb wave wavenumber-searching method for a linear PZT sensor array. The method expands the applicability of the spatial-wavenumber-domain-based damage imaging and localization methods, because it only requires Nyquist sampling theorem satisfaction by the temporal sampling frequency but not by the spatial sampling rate. A validation experiment of the proposed Lamb wave wavenumber-searching method is implemented on a 2024-T3 aluminum alloy and compared with the results obtained from a classical structural model technique. The validation results showed good wavenumber calculation accuracy with a maximum wavenumber calculation error of no more than 2.2 rad/m. The Lamb wave wavenumber-searching method-based damage localization method is also validated on a carbon fiber composite laminate plate. The validation results showed good damage localization accuracy with errors of no more than 2.11 cm.

However, the proposed method is only usable for a single mode of a narrowband-frequency Lamb wave, and its principle is based on the linear PZT sensor array and far-field propagation. The group velocity of the Lamb wave is also needed to calculate damage distance. These aspects limit the performance and applicability of damage location based on the proposed method. Thus, further work is ongoing to study these problems, as well as to investigate the validation of the proposed method in more situations and structures.

## Figures and Tables

**Table 1:** Mechanical properties of the 2024-T3 aluminum alloy.

Parameter	Value
Density / (kg·m^-3^)	2.78×10^3^
Elastic modulus / GPa	73.1
Shear modulus / GPa	28
Poisson ratio *µ*	0.33

**Table 2:** Theoretical wavenumber of *A*_0_ mode calculated by the Rayleigh–Lamb equation solving method.

Frequency (kHz)	Theoretical Wavenumber (rad/m)
30	261.67
35	283.63
40	304.26
45	323.83
50	342.53
55	360.47
60	377.78
65	394.54
70	410.82

**Table 3:** The wavenumber of each actuator and central frequency calculated by the Lamb wave wavenumber-searching method.

Signal Source	Frequency (kHz)	Theoretical Wavenumber (rad/m)	Lamb Wave Wavenumber-Searching Method (rad/m)	Wavenumber Error (rad/m)
PZT A	30	261.67	259.8	−1.9
	35	283.63	282.3	−1.3
	40	304.26	302.5	−1.8
	45	323.83	325.3	1.5
	50	342.53	340.4	−2.1
	55	360.47	358.8	−1.7
	60	377.78	375.6	−2.2
	65	394.54	393.1	−1.4
	70	410.82	408.8	−2.0
PZT B	30	130.84	131.5	0.7
	35	141.82	142.6	0.8
	40	152.13	153.6	1.5
	45	161.92	161.7	−0.2
	50	171.27	171.2	−0.1
	55	180.24	179.9	−0.3
	60	188.89	188.2	−0.7
	65	197.27	196.8	−0.5
	70	205.41	204.9	−0.5
PZT C	30	−168.20	−166.3	1.9
	35	−182.31	−180.6	1.7
	40	−195.57	−193.7	1.9
	45	−208.15	−206.9	1.3
	50	−220.17	−218.2	2.0
	55	−231.71	−231.0	0.7
	60	−242.83	−242.0	0.8
	65	−253.61	−252.5	1.1
	70	−264.07	−262.1	2.0

**Table 4:** Material parameters of a single layer of the composite plate.

Parameter	Value
0° tensile modulus (GPa)	135
90° tensile modulus (GPa)	8.8
± 45° in-plane shearing modulus (GPa)	4.47
Poisson ratio *µ*	0.328
Density (kg·m^-3^)	1.61 × 10^3^

**Table 5:** Positions of the simulated damages and the reference PZT sensors.

Position Label	Cartesian Coordinates (mm, mm)	Polar Coordinates (°, mm)
Ref 1	(200, 0)	(0.0, 200.0)
Ref 2	(200, 200)	(45.0, 282.8)
Ref 3	(0, 400)	(90.0, 400.0)
A	(100, 300)	(71.6, 316.2)
B	(100, 200)	(63.4, 223.6)
C	(0, 200)	(90.0, 200.0)
D	(−100, 200)	(116.6, 223.6)
E	(−200, 200)	(135.0, 282.8)
F	(−200, 100)	(153.4, 223.6)
G	(−200, 0)	(180.0, 200.0)

**Table 6:** Damage localization results of the seven damages.

Damage Label	*k_n_*_1_ (rad/m)	*k_n_*_2_ (rad/m)	Arrive Time (ms)	Start Time (ms)	Localized Position (mm, mm)	Actual Position (mm, mm)	Damage Localization Error (mm)
A	121.5	328.3	0.5124	0.1032	(109.2, 295.0)	(100, 300)	10.4
B	163.1	299.8	0.4094	0.1034	(112.4, 206.6)	(100, 200)	14.1
C	4.8	340.3	0.3583	0.1034	(2.8, 195.9)	(0, 200)	4.9
D	−152.4	304.7	0.3861	0.1034	(−97.2, 194.4)	(−100, 200)	6.3
E	−241.7	228.9	0.4570	0.1034	(−197.4, 186.9)	(−200, 200)	13.4
F	−309.2	151.5	0.4001	0.1031	(−205.0, 100.5)	(−200, 100)	5.0
G	−343.1	−9.3	0.3897	0.1031	(−220.0, −6.0)	(−200, 0)	21.1
